# Mechanical Property Characterization of Virgin and Recycled PLA Blends in Single-Screw Filament Extrusion for 3D Printing

**DOI:** 10.3390/polym16243569

**Published:** 2024-12-20

**Authors:** Reem Aly, Olafisoye Olalere, Aaron Ryder, Mozah Alyammahi, Wael A. Samad

**Affiliations:** 1 Department of Mechanical & Industrial Engineering, Rochester Institute of Technology, Dubai 341055, United Arab Emirates; rha3005@rit.edu (R.A.); oco3885@rit.edu (O.O.); 2 Department of Mechanical Engineering, Rochester Institute of Technology, Rochester, NY 14623, USA; atr5272@rit.edu; 3 R&D Technologist Dubai Electricity & Water Authority, Dubai 341055, United Arab Emirates; mozah.alyammahi@dewa.gov.ae

**Keywords:** 3D printing, recycling, extrusion, mechanical testing, filament making, polylactic acid

## Abstract

Additive manufacturing is an attractive technology due to its versatility in producing parts with diverse properties from a single material. However, the process often generates plastic waste, particularly from failed prints, making sustainability a growing concern. Recycling this waste material presents a potential solution for reducing environmental impact while creating new, functional parts. In this study, the feasibility of creating printable filaments from recycled polylactic acid (PLA) waste and virgin PLA pellets was explored. Filaments were manufactured in the lab using a single-screw desktop extruder with four temperature zones, with compositions ranging from 100% virgin PLA to 100% recycled PLA in 10% composition increments. Test samples were 3D printed using a Material Extrusion 3D printer and subjected to tensile testing in conjunction with digital image correlation to evaluate their ultimate tensile strength, yield strength, Young’s modulus, ductility, toughness, and strain distribution. The results indicated that the optimal mechanical properties were observed in specimens made from 100% virgin PLA, 100% recycled PLA, and a 50% virgin/50% recycled PLA blend. Additionally, comparisons with a commercially produced PLA filament revealed that 100% virgin and 100% recycled blends have a 50.33% and 48% higher tensile strength than commercial filament, respectively. However, commercial filaments exhibited higher ductility and toughness than the lab-made extruded filament.

## 1. Introduction

3D printing technology has evolved significantly from producing simple plastic products in the early 2000s to applications in a wide range of sectors today [1]. During the COVID-19 pandemic, additive manufacturing played a crucial role in producing large quantities of medical discharge devices and masks [1]. Despite its benefits, 3D printing waste has been growing at a rapid pace, with an annual increase of 26% in waste production [2]. By 2022, the UK alone had 132,000 3D printers, consuming approximately 1,148,400 kg of filament per year [3]. Surveys from 2019 revealed that between 6% and 19% of this material is wasted, contributing to 379,000 kg of waste filament ending up in landfills annually [4]. Given that PLA takes over 80 years to decompose, this waste adds significantly to environmental pollution [5]. The primary causes of 3D printing waste include test prints, unwanted prototypes, support structures, and failed prints [4].

Efforts to reduce waste have led researchers to explore the potential of recycling PLA filaments. Oladapo et al. examined the possibility of achieving net-zero waste in additive manufacturing, finding that producing recycled filament consumes less energy than creating virgin PLA, which results in lower greenhouse gas emissions. Additionally, recycling through pyrolysis can reduce energy consumption by 60% [6]. Bergaliyeva et al. investigated the recycling of PLA waste printed using FFF technology by shredding, drying, and mixing the shreds with 25%, 50%, and 75% content of virgin PLA before extrusion [7]. The results indicated that as the recycled content increased, the samples went from brittle to more ductile in their characteristics. The 25–75 virgin-PLA blend has the highest tensile strength due to having smaller voids, reduced micro-pore density, higher melt flow index and an overall better inter-layer bonding. Similarly, Agbakoba et al. explored the recyclability of PLA bio-composites, showing that a 20% waste PLA blend with 80% virgin PLA increased ultimate tensile strength (UTS) and elongation compared to 100% virgin PLA [8]. The study also found that fully recycled PLA exhibited higher stiffness due to enhanced crystallization and shorter polymer chains, closely matching the mechanical properties of commercially available PLA filaments.

While tensile tests offer valuable insights, Anderson expanded the scope by conducting tensile, shear, and hardness tests on both virgin and recycled PLA [2]. Their results indicated minimal deviation in mechanical properties, with recycled PLA showing a slight decrease in tensile strength and hardness but an increase in shear strength. Patti et al. further investigated the differences between virgin and recycled PLA, revealing that recycled PLA has reduced molecular weight, thermal instability, and lower viscosity, although mechanical behavior remained relatively consistent under certain conditions [9]. Several researchers have focused on enhancing the extrusion process for recycled PLA. Babagowda et al. studied the production of recycled PLA pellets from sources like bottles and containers, finding that a 10% recycled composition with a 0.1 mm layer thickness improved tensile strength, while a 20% recycled composition enhanced flexural strength [10]. Oussai et al. investigated the recyclability of PET, showing increased tensile and shear strength but reduced elongation and hardness in recycled PET, though extrusion and printing challenges persisted [11]. Gonzalez et al. also explored recycling PET and HDPE from plastic water bottles into functional 3D-printed products, demonstrating the potential of recycled materials in fused granular fabrication [12].

Romani et al. studied the effects of multiple recycling cycles on PLA feedstock waste, observing that key mechanical properties like elastic modulus and UTS decreased with each cycle, with a 20% reduction in UTS by the fifth cycle [13]. Similarly, Hidalgo-Carvajal et al. investigated recycling PLA waste from PPE, finding significant declines in molecular weight and tensile strength after three extrusion cycles [14]. Daniel et al. examined the addition of virgin PLA to recycled PLA, noting that while a 75% virgin PLA blend improved strength, further research is needed to fully understand these effects [15]. Lanzotti et al. conducted three-point bending tests on virgin and recycled PLA, finding that the strength of virgin PLA decreased after three extrusion cycles, with a significant drop occurring after the third cycle [16]. Cruz Sanchez et al. compared four different recycling methods and concluded that mechanical properties, including tensile strength and strain, degraded significantly after five recycling cycles, impairing the performance of 3D-printed recycled filament [17]. Beltran et al. studied methods to increase the molecular weight of recycled PLA, using Solid-State Polymerization to restore some of the mechanical properties lost during recycling [18].

Recycling polymers is an actively wide research field. Previous papers investigate the incorporation of additives in recycled plastics to produce viable 3D printing filaments with the aim of achieving a circular economy. Ghabezi et al. experimented with recycling polypropylene, plastic mushroom containers, into 3D printing filament with small fractions of basalt fiber to enhance its mechanical properties [19]. It was observed that the addition of 2% of basalt fibers enhanced both the printability and mechanical properties. Adding 15% basalt fiber resulted in the highest tensile strength and 30% provided the best flexural strength; however, adding more led to a degradation of the properties. Similarly, Sam-Daliri et al. attempted to investigate the recyclability of polypropylene and glass fiber-reinforced polypropylene with the addition of glass fiber by testing its performance at different percentages [20]. When 15% of glass fiber was added to recycled glass fiber-reinforced polypropylene, the UTS increased by 275% when compared with pure polypropylene. This study proved how with good-quality filament production, the mechanical properties can be greatly improved by the incorporation of glass fiber into polymers. Research by Wu et al. explored how recycled discontinuous carbon fibers evolve throughout the filament production and printing process to observe the examine the relationship between fiber length, fiber orientation, and mechanical behavior [21]. A range of recycled carbon fiber lengths were mixed with polyamide-6. It was concluded that the fiber lengths do decrease during the extrusion due to the shear forces, however the longer the lengths and right-oriented the fibers are, the higher the tensile strength and Young’s modulus is.

Efforts to improve filament production include optimizing the extrusion process for consistent flow and uniform filament diameter using a desktop single-screw extruder [22]. Finally, Bakhtiari et al. reviewed factors affecting fatigue properties in Fused Filament Fabrication (FFF), identifying raster angles, infill patterns, and build orientations as key contributors to stress concentrations. They also studied how recycled content affects these stress concentrations in 3D-printed PLA [23].

The objective of this study was to produce filaments by blending virgin PLA pellets with shredded waste PLA parts, followed by the fabrication of test specimens according to ASTM D638 Type 1 for mechanical property evaluation. A total of 11 test samples were printed, ranging from 100% virgin PLA to 100% recycled PLA, with 10% increments in composition between each set. The specimens were tested using a universal testing machine in conjunction with digital image correlation (DIC) to analyze strain profiles and distributions on the surface of the specimens. This study is particularly relevant for 3D printing enthusiasts and university labs who enjoy the benefits of additive manufacturing but often accumulate failed prints.

## 2. Experimental Methods

**Figure 1:**
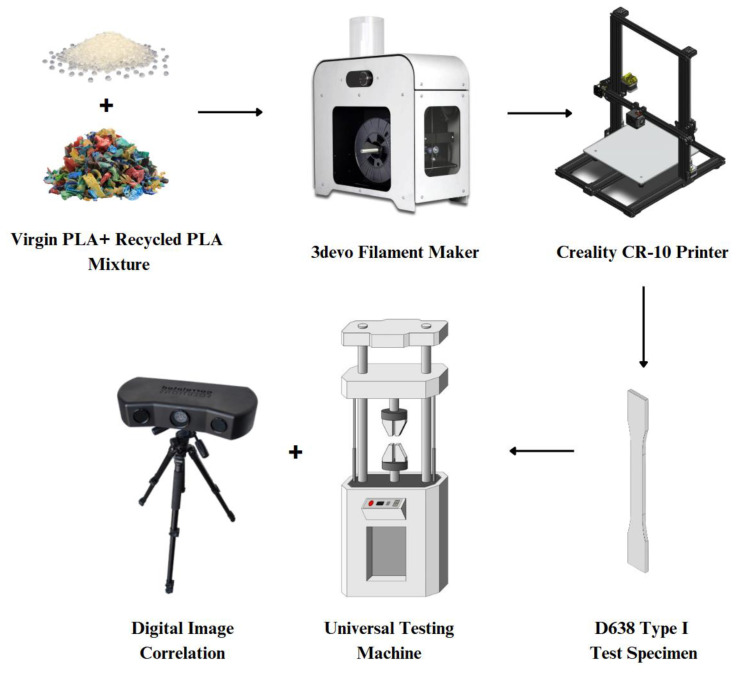
Schematic diagram of the PLA upcycling process by using material extrusion filament and 3D printing techniques.

In this study, PLA 3D prints were collected, shredded, and mixed with virgin PL pellets, then extruded into new filament spools for subsequent printing. All filaments and test specimens were produced using a desktop-sized single-screw filament extruder by 3devo (Utrecht, The Netherlands) and an FDM 3D printer by Creality (Shenzhen, China). The specimens, with compositions ranging from 100% virgin PLA to 100% recycled PLA, underwent tensile testing in conjunction with DIC. Additionally, the properties from all composition variations were compared and benchmarked against that of a commercially available PLA filament from Wanhao (Zhejiang, China). Figure 1 provides a schematic overview of the experimental procedure and the steps involved in this study.

### 2.1. Material Collection

Over an academic year, numerous print jobs were carried out on FDM printers on campus. These print runs inevitably resulted in some failed prints, excess parts, support structures, and filament transitions, all of which were discarded. For this study, all waste 3D-printed PLA, regardless of color or shape, was collected throughout the year for use in recycled filament production in this particular study. Figure 1 provides a schematic overview of the experimental procedure and the steps involved in this study.

To prepare the waste material for extrusion, it was essential to shred the collected PLA prints into particles no larger than 4 mm in diameter. A mini shredder equipped with an NRV050 reducer (Zhejiang, China) was used for this process. The waste prints were passed through the shredder for 4 to 6 cycles, followed by sieving using a stainless steel sifter with a 3 mm mesh to separate smaller particles. Eleven different mixtures of recycled PLA shreds and virgin PLA pellets were prepared for extrusion, with each batch weighing 200 g to ensure sufficient filament for sample printing, while accounting for trial and error. The mixtures were precisely measured using a Citizen CZ 602 scale (Tokyo, Japan) with two-decimal-point accuracy. Table 1 presents the composition of the eleven mixtures, detailing the percentages of recycled (R) and virgin (V) PLA in each batch. Throughout this manuscript, “R” will refer to recycled PLA shreds and “V” will refer to virgin PLA pallets.

### 2.2. Filament Making

**Figure 2:**
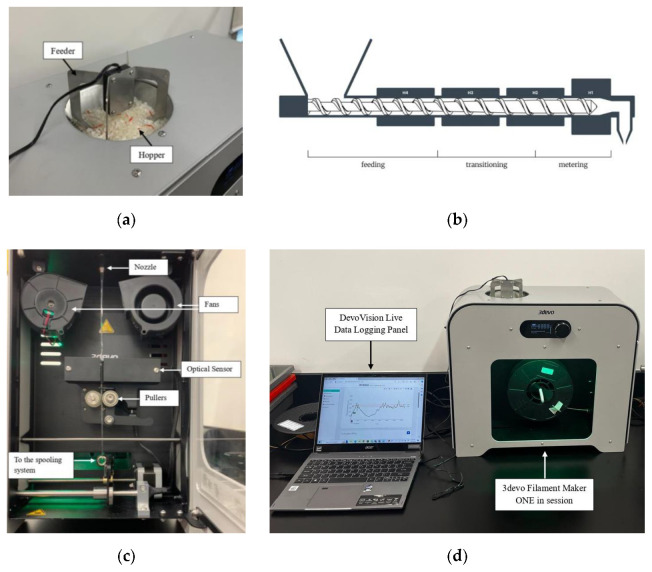
(**a**) Feeder placed at the hopper inlet. (**b**) Schematic of the zones in the single screw 3devo Filament Maker [25]. (**c**) Filament maker schematic. (**d**) Live data logging from the 3devo Filament Maker ONE Composer.

After the waste prints were shredded, sieved to remove larger particles, and roughly mixed with virgin PLA in the appropriate percentage weight compositions, the next step was extruding the mixture into filament spools for subsequent 3D printing. This process was carried out using the Filament Maker ONE Composer from 3devo, a desktop-sized system equipped with a single-screw extruder, four heating zones, a cooling system with dual fans, an optical sensor for diameter control, and a spooling mechanism. The extruder is divided into three zones—feeding, transition, and melting. The extruder screw is an advanced mixing screw that helps to ensure that various additives or polymers are well-mixed with the base plastic before exiting the nozzle. In our case, it assists in evenly mixing the PLA shreds with the virgin pellets, making a well-blended spool. The PLA mixture, consisting of both pellets and shredded material, was poured into the hopper, which introduced a degree of heterogeneity to the mixture. To ensure a consistent feed into the extruder, a feeder (Figure 2a) was installed in the hopper. The feeder pulsed at one-second intervals, vibrating the mixture and providing steady input to the feeding zone. The four heaters were then preheated to the previously set temperatures, with Heater 4 located at the entry point and Heater 1 at the exit, as shown in Figure 2b. The temperatures of all four heaters, extruder RPM, and fan speed were set according to 3devo guidelines, as these settings differ between virgin PLA pellets and non-dried PLA shreds [24]. The temperatures provided for the non-dried PLA shreds were also set for the extrusion of mixtures of PLA pellets and PLA shreds. Table 2 presents the temperature settings for each heater, along with the fan speeds and screw speed parameters used to produce all 11 spools of virgin and recycled PLA filament.

**Figure 3:**
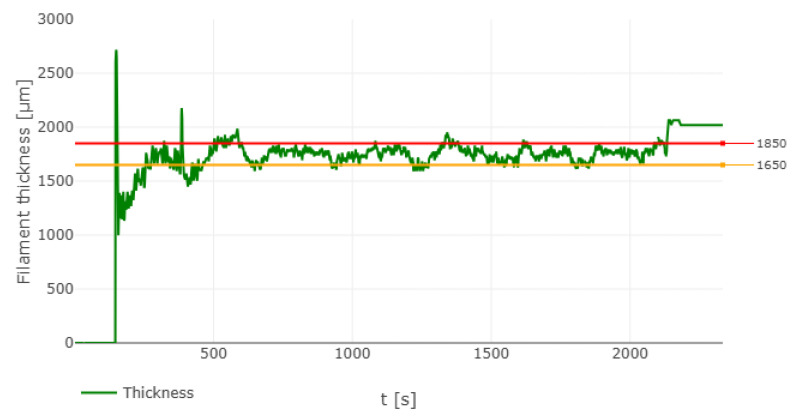
Filament thickness of 50%V/50% R PLA from DevoVision.

In preparation for the filament extrusion process, key parameters were configured in the extruder, including preheating the screw and setting the dimensions of the spool. Critical measurements, such as the spool’s width and its diameter when full and empty, were input into the system to ensure the spool mechanism maintained appropriate tension during winding. These measurements were obtained using a vernier caliper. The desired filament diameter was set to 1.74 mm, closely matching that of commercially available filaments. Once all parameters were configured and the screw reached the required preheat temperature, the extrusion process commenced. As the filament exited the nozzle and passed between cooling fans, it was monitored by an optical sensor before traveling through two pulleys, which controlled its thickness by adjusting their relative speeds. Figure 2c provides a labeled illustration of the nozzle, fans, optical sensor, and puller wheels. The filament was then wound onto the spool for subsequent use in 3D printing. The 3devo extruder offers live data-logging capabilities via its DevoVision software v2.0. The software continuously logs critical parameters such as heater temperatures, extruder RPM, filament diameter, fan speed, and total filament length. These logs are saved after each extrusion session for further analysis. Figure 2d shows the setup used for live data logging. Additionally, Figure 3 illustrates the filament thickness of a spool composed of 50% virgin PLA and 50% recycled PLA, with lower and upper bounds set at 1.65 mm and 1.85 mm, respectively.

### 2.3. Three-Dimensional Printing and Testing

**Figure 4:**
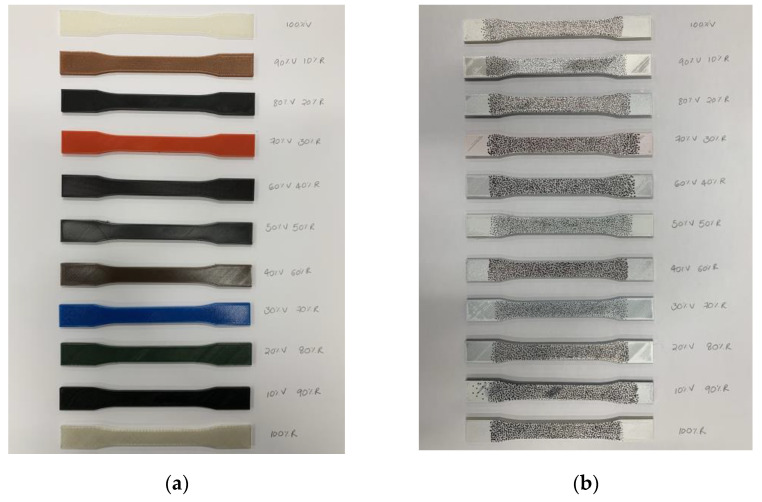
(**a**) All 3D printed samples. (**b**) Samples prepared for DIC testing.

Following ASTM D638 standards for the tensile testing of plastics [26], a Type I specimen was designed using Autodesk Inventor Professional 2023. The STL file was subsequently exported and uploaded to UltiMaker Cura to adjust the print settings. Table 3 details the print specifications applied to all samples for subsequent mechanical testing. Two specimens were arranged on the print bed using UltiMaker Cura, and the slicing process estimated a print time of approximately 1 h and 50 min. The printing was performed using a Creality CR-10 3D printer. Figure 4a shows the completed printed samples that were prepared for further testing.

**Figure 5:**
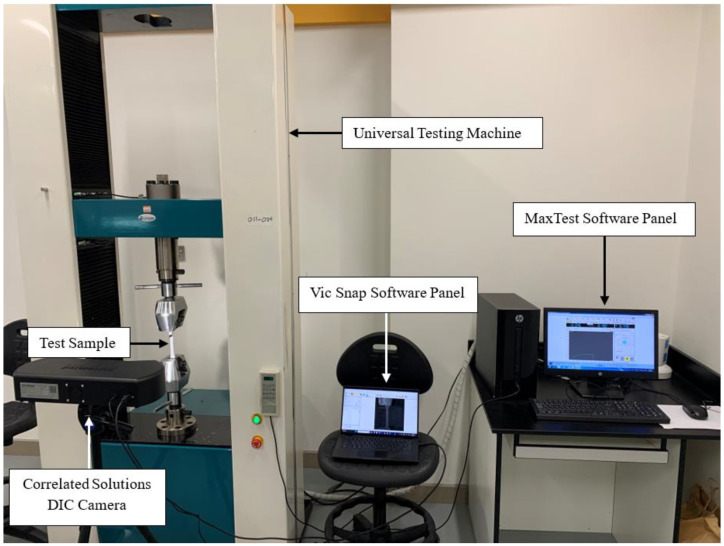
Overall testing setup.

After 3D printing two specimens for each percentage composition, the samples were prepared for simultaneous tensile testing and DIC. The specimens were first spray-painted white using Asmaco high-gloss spray paint. Once dried, black speckle patterns were manually applied across the surface of each specimen using a Pilot Sca-Tm-B black marker. Figure 4b displays all 3D-printed samples ready for DIC testing. Tensile testing was conducted using a servo motor-driven universal testing machine (UTM) from Laryee Technology (Beijing, China), with the uniaxial tensile test parameters controlled through MaxTest software also by Laryee Technology. For the DIC tests, a Correlated Solutions (South Carolina, USA) system was used, with VicSnap software for image acquisition and Vic3D software for image correlation and post-processing also by Correlated Solutions (South Carolina, USA). Each specimen was securely gripped in the UTM to prevent slippage during testing. The DIC camera was adjusted for optimal focus, brightness, and polarizing filter settings, followed by calibration. Once the setup was complete, the tensile test commenced, and the DIC camera simultaneously captured images at 4 s intervals, resulting in a total of 50 images. The tensile testing machine operated at a constant crosshead speed of 1 mm/minute. Figure 5 illustrates the overall setup for this testing procedure. This process was repeated for all specimens across the different composition percentages. Data were recorded and saved for further analysis, including extracting the desired mechanical properties. The images captured by Vic Snap were imported into Vic3D for correlation, while the text files from the MaxTest software were imported into MATLAB R2023a for analysis and plotting.

## 3. Results and Discussion

Two test specimens were 3D printed for each percentage composition set. It is worth noting that a few challenges were encountered while printing two of the eleven sets, primarily due to inconsistent extruded filament diameter, which led to print failures. Table 4 lists all the tested samples and the corresponding set names assigned to their data.

### 3.1. Tensile Testing

**Figure 6:**
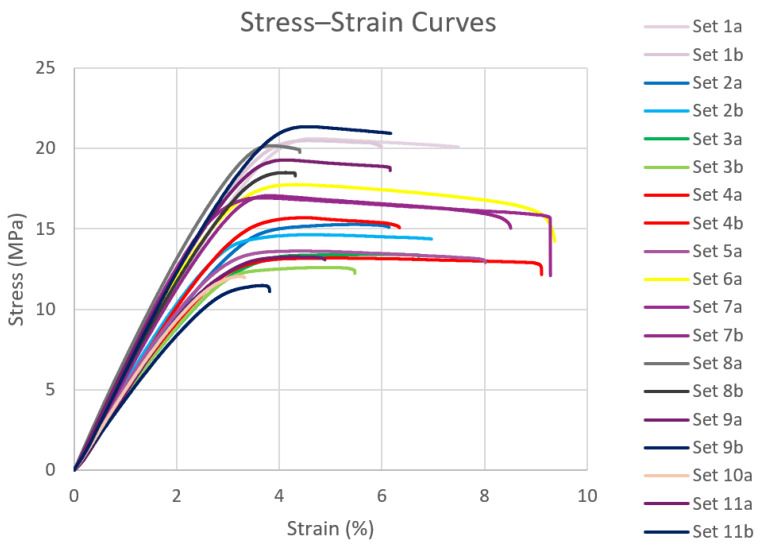
Stress vs. strain plot for all tested samples.

**Figure 7:**
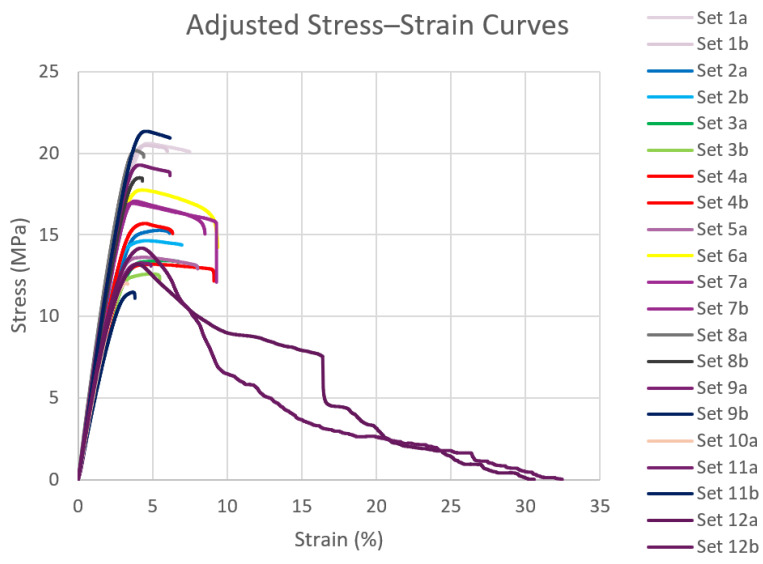
Stress vs. strain comparison of all samples with commercial PLA.

**Figure 8:**
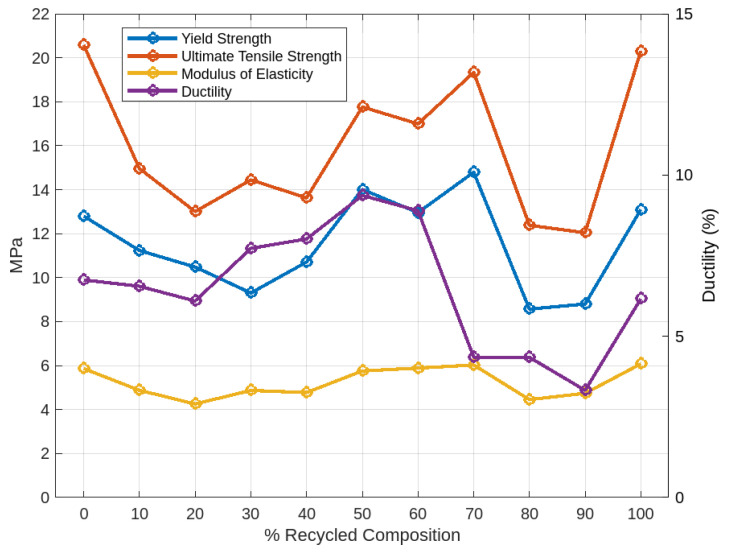
Yield, UTS, elastic modulus, and ductility across all samples.

**Figure 9:**
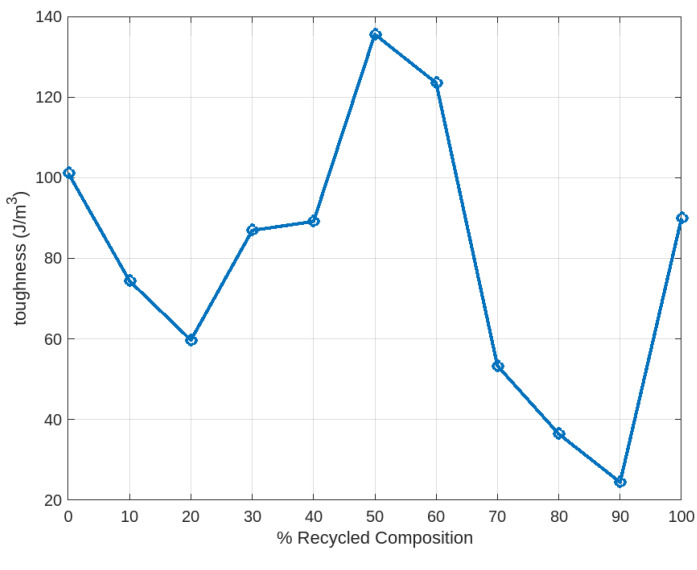
Toughness across all samples.

After conducting the tensile tests for all samples, stress–strain data were collected and imported into MATLAB for further analysis. The stress–strain curves for all samples, obtained from the tensile testing, are presented in Figure 6. For comparison with the filaments produced using the 3devo Filament Maker, an additional test specimen was 3D printed using a commercially available filament. Its stress–strain curve is shown in Figure 7, alongside those of the other test samples. From the plotted stress–strain curves, key mechanical properties such as yield strength, ultimate tensile strength, elastic modulus, ductility, and toughness were extracted and evaluated for both the 3devo-produced and commercial PLA (C-PLA) samples. For sets with two test specimens, the values were averaged and are summarized in Table 5. Figure 8 and Figure 9, plotted using MATLAB, present the values for each property across all samples.

It is worth noting that sets 12a and 12b in Figure 8 suggest a ductility value exceeding 30%. However, the actual values used were 17% and 8.5%, respectively, reflecting the point of failure and the significant drop in the measured stress from the UTM’s load cell. Although the specimens continued to elongate as the crosshead pulled them further, they had effectively failed beyond those strain values.

The stress–strain curves in Figure 6 of all lab-extruded PLA samples exhibited typical tensile behavior for PLA materials. A quick examination of Figure 8 and Figure 9 reveals that superior mechanical properties are observed at the extremes and center of the composition range. Specifically, the 100% virgin PLA, 100% recycled PLA, and 50% virgin/50% recycled PLA blends demonstrated the most favorable properties. This superior performance is attributed to the uniformity of the mixtures, ensuring consistent material distribution during the filament extrusion process. Single-screw desktop extruders, like the one used in this study, perform best with feedstock of uniform size and geometry. While set 11 (100% recycled PLA) consisted of shreds or flakes rather than pellets, it still benefited from consistency, resulting in good overall extrusion performance.

In contrast, compositions such as 80% virgin/20% recycled and 10% virgin/90% recycled, for instance, exhibited poorer mechanical properties due to the irregular mixing of shreds and pellets. In these cases, the minority component acted as an impurity in a host material. This phenomenon was reflected in the degradation of mechanical properties. One potential solution to improve the uniformity of these mixtures would be to subject the filament to multiple extrusion cycles. Re-extruding the filament after the initial extrusion could enhance material homogeneity. However, this approach would likely result in the degradation of mechanical properties, as observed in other studies after multiple extrusion cycles. Additionally, from the perspective of hobbyists or small-scale labs, repeated extrusion may not be practical due to the increased complexity and equipment limitations involved.

Taking a closer look at the properties in Figure 8, but in tabulated form revealing the percentage difference to the benchmark commercial PLA, revealed that the 100% virgin set showed significant improvements in key properties, including a 50.33% increase in UTS and an 18.86% increase in modulus, at the expense of ductility. Similar improvements were also seen in the 100% recycled set, where the UTS improved by 48% at the expense of a 46% reduction in ductility. Table 6 also includes a color scale, ranging from dark green for high positive percentage change to dark red for negative percentage change. It is not uncommon for filaments produced using desktop single-screw extruders to exhibit significant reductions in ductility compared to commercial ones. This is primarily due to the more controlled industrial production processes, which utilize cooling systems such as temperature-regulated water baths, unlike the fans used in the 3devo extruder. Factors such as cooling rate, extrusion speed, and moisture content likely contributed to the inferior performance of lab-extruded filament.

Reflecting on the previous literature, it was similarly concluded that recycled PLA filaments tend to have higher mechanical properties in comparison with virgin PLA. A PLA blend with 75% recycled 25% virgin PLA increased the tensile strength by 19%, which was justified due to the smaller voids and better inter-layer bonding between the 3D printer layers observed in the SEM tests in contrast with the bigger voids on the pure PLA prints [7]. Another study used waste 3D prints from Esun black filament, and the 100% recycled filament had a UTS of 34.4 MPa while 100% virgin had 31.6 MPa; this increase in the stiffness is due to the increase in crystallization and shortening of the polymer chains [8].

### 3.2. Digital Image Correlation

**Figure 10:**
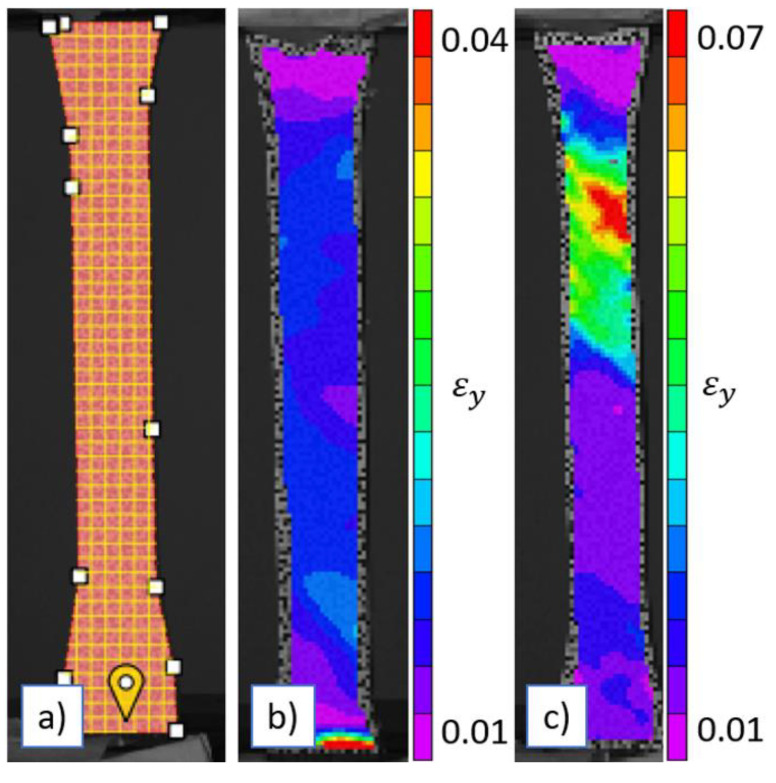
(**a**) DIC area of interest, (**b**) vertical strain at halfway mark (50%V/50%R), and (**c**) vertical strain just before failure (50%V 50%R).

As previously mentioned, all uniaxial tensile tests were conducted in conjunction with DIC. To eliminate potential biases, all tests were performed under consistent ambient conditions, including temperature, humidity, and lighting. Furthermore, the same set of calibration images was used across all tests to ensure uniformity in post-processing and strain field extraction. DIC images were captured using VIC Snap software, and the subsequent image correlation was conducted using VIC 3D, both provided by Correlated Solutions, Inc. (Columbia, South Carolina, USA) Figure 10a illustrates the DIC area of interest on the 3D printed samples, Figure 10b shows the distribution of the vertical strain at halfway through the tensile testing of 50%V 50%R and lastly, Figure 10c portrays the vertical strain just before failure for the 50%V/50%R.

The 50% virgin/50% recycled blend achieved an optimal balance between performance and sustainability. The homogeneity of the composition, with no impurity/host material effect due to the equal ratio of virgin and recycled PLA, contributed to this balance. The DIC results, shown in Figure 10, further supported this finding, as the strain contours revealed a uniform vertical strain profile, indicating a smooth surface finish on the 3D-printed samples. This uniformity was also reflected in the diameter vs. time data, presented in Figure 3. The blend demonstrated moderate improvements in ultimate tensile strength and modulus of elasticity, with less pronounced reductions in ductility and yield stress compared to blends with higher recycled content. Consequently, the 50% virgin /50% recycled blend represents an optimal choice for applications where both mechanical performance and environmental sustainability are critical considerations.

The primary objective of this study was to evaluate the feasibility of recycling waste PLA prints and to identify optimal filament compositions for 3D printing. The findings demonstrate that the 100% virgin, 100% recycled, and 50% virgin/50% recycled PLA blends exhibit the best mechanical properties. In particular, the 50% virgin/50% recycled blend offers a promising balance between performance and sustainability, making it an ideal choice for applications requiring both mechanical strength and environmental benefits. Ensuring proper feedstock preparation is essential for maintaining consistent filament diameter and printability. Early challenges related to inconsistent shred sizes were effectively addressed using a 3 mm sieve, which improved material mixing and produced more uniform filaments. Future research should focus on optimizing sieve sizes and extruder specifications to further enhance filament quality and consistency. This will enable more reliable production of high-quality, printable filaments from recycled PLA, making 3D printing more sustainable for hobbyists and small-scale users alike.

## 4. Conclusions

The practice of 3D printing continues to evolve as a transformative technology in additive manufacturing, yet challenges such as failed print jobs contribute to significant waste and material inefficiency. This study addressed an aspect of this issue by investigating the potential of recycling PLA waste into new filaments using an accessible, single-screw desktop extruder. By targeting 3D printing enthusiasts and university labs facing excess PLA waste, the research evaluated filaments with increasing recycled PLA content, ranging from 0% to 100%. Mechanical testing in conjunction with DIC revealed that filaments with 100% recycled PLA and a 50/50 blend of virgin PLA pellets and recycled PLA shreds exhibited superior ultimate tensile strength and yield strength, with some trade-offs in ductility when compared with commercial PLA filament. The 100% recycled PLA had a 48% increase in its ultimate tensile strength which then led to a 41.3% drop in its ductility in comparison with c-PLA and similar behaviors were observed with 100% virgin pellets and the 50/50 mixture of both. These results suggest that recycled PLA can be effectively repurposed into high-performance filaments for FDM printers, offering a cost-effective and sustainable solution for a wide range of applications.

## Figures and Tables

**Table 1:** Filament composition of virgin and recycled PLA.

Mixture Content [%]	Mass of Virgin PLA Pallets [g]	Mass of Recycled PLA Shreds [g]
100%V 0%R	200	0
90%V 10%R	180	20
80%V 20%R	160	40
70%V 30%R	140	60
60%V 40%R	120	80
50%V 50%R	100	100
40%V 60%R	80	120
30%V 70%R	60	140
20%V 60%R	40	160
10%V 90%R	20	180
0%V 100%R	0	200

V stands for virgin PLA; R stands for recycled PLA.

**Table 2:** 3devo Filament Maker extrusion settings.

	Heater 1	Heater 2	Heater 3	Heater 4	Screw Speed	Fan Speed
100% Virgin PLA	170 °C	190 °C	185 °C	170 °C	3.5 RPM	80%
Mixtures with shreds	180 °C	185 °C	190 °C	180 °C	5 RPM	70%

**Table 3:** **Three-dimensional** printing settings.

Infill Density	Infill Pattern	Nozzle Temperature	Bed Temperature
40%	Grid	210 °C	60 °C

**Table 4:** Corresponding set name for each sample.

Set Name	Test Sample Content	Set Name	Test Sample Content
Set 1a	100%V 0%R	Set 7a	40%V 60%R
Set 1b		Set 7b	
Set 2a	90%V 10%R	Set 8a	30%V 70%R
Set 2b		Set 8b	
Set 3a	80%V 20%R	Set 9a	20%V 80%R
Set 3b		Set 9b	
Set 4a	70%V 30%R	Set 10a	10%V 90%R
Set 4b		Set 11a	0%V 100%R
Set 5a	60%V 40%R	Set 11b	
Set 5b		Set 12a	100%C-PLA
Set 6a	50%V 50%R	Set 12b	

**Table 5:** Summary of material properties.

Set Name	Yield Stress [MPa]	UTS [MPa]	Modulus [GPa]	Ductility, %EL [%]	Toughness [J/m3]
100%V 0%R	12.80	20.58	5.86	6.75	101.13
90%V 10%R	11.23	14.96	4.88	6.55	74.39
80%V 20%R	10.49	13.01	4.26	6.09	59.72
70%V 30%R	9.30	14.45	4.87	7.73	86.94
60%V 40%R	10.71	13.63	4.78	8.02	89.15
50%V 50%R	14.02	17.77	4.76	9.37	135.57
40%V 60%R	12.96	17.00	5.89	8.90	123.51
30%V 70%R	14.80	19.34	6.03	4.35	53.33
20%V 60%R	8.57	12.38	4.45	4.35	36.44
10%V 90%R	8.80	12.04	4.74	3.32	24.46
0%V 100%R	13.10	20.31	6.08	6.17	89.96
C-PLA	12.80	13.69	4.93	31.53	169.78

**Table 6:** Percentage change in mechanical property with respect to commercial PLA.

Set Name	Yield Stress	UTS	Modulus	Ductility
100%V 0%R	0.0	+50.3	+18.9	−41.3
90%V 10%R	−12.3	+9.3	−1.0	−43.0
80%V 20%R	−18.0	−5.0	−13.6	−47.0
70%V 30%R	−27.3	+5.6	−1.2	−32.8
60%V 40%R	−16.3	−0.4	−3.0	−30.3
50%V 50%R	+9.5	+29.8	+16.8	−18.5
40%V 60%R	+1.3	+24.2	+19.5	−22.6
30%V 70%R	+15.6	+41.3	+22.3	−62.2
20%V 60%R	−33.0	−9.6	−9.7	−62.2
10%V 90%R	−31.3	−12.1	−3.9	−71.1
0%V 100%R	+2.3	+48.4	+23.3	−46.3

## Data Availability

The data presented in this work are available upon request from the corresponding authors.
